# Supplementary figures and images for: Babesia bovis Rad51 ortholog influences switching of ves genes but is not essential for segmental gene conversion in antigenic variation
Source: PLoS Pathog. 2020 Aug 31;16(8):e1008772. doi: 10.1371/journal.ppat.1008772 (PMC7485966; doi:10.1371/journal.ppat.1008772)

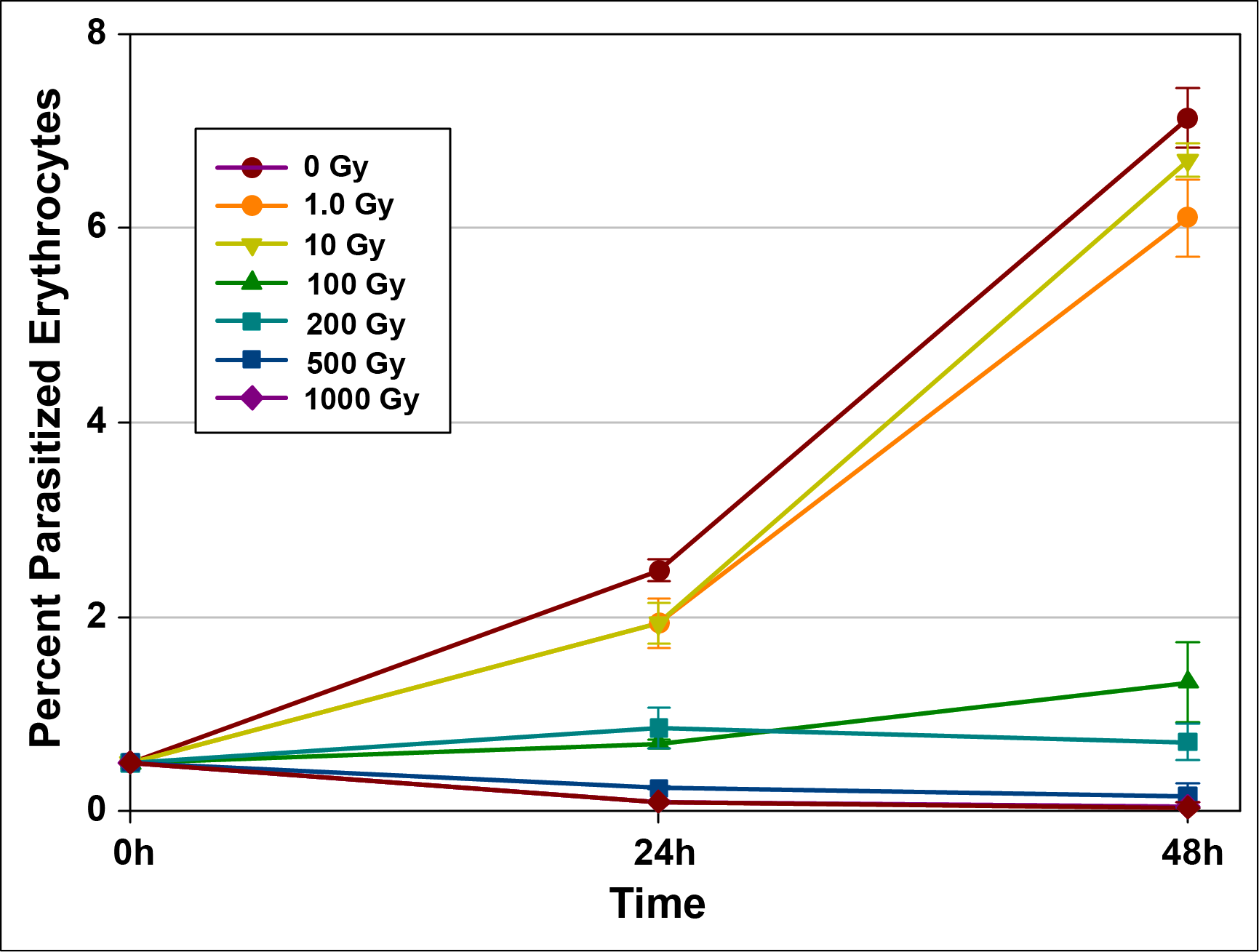

Supplement: S1 Fig — Parasites were exposed to 0–1000 Gy irradiation from a calibrated Cs137 source, then placed back into culture and the percent parasitized erythrocytes determined from Giemsa-stained smears made at 0, 24, and 48h growth. For experiments requiring survival of a proportion of the parasites the 100 Gy dosage was chosen. (TIF) [file ppat.1008772.s001.tif]

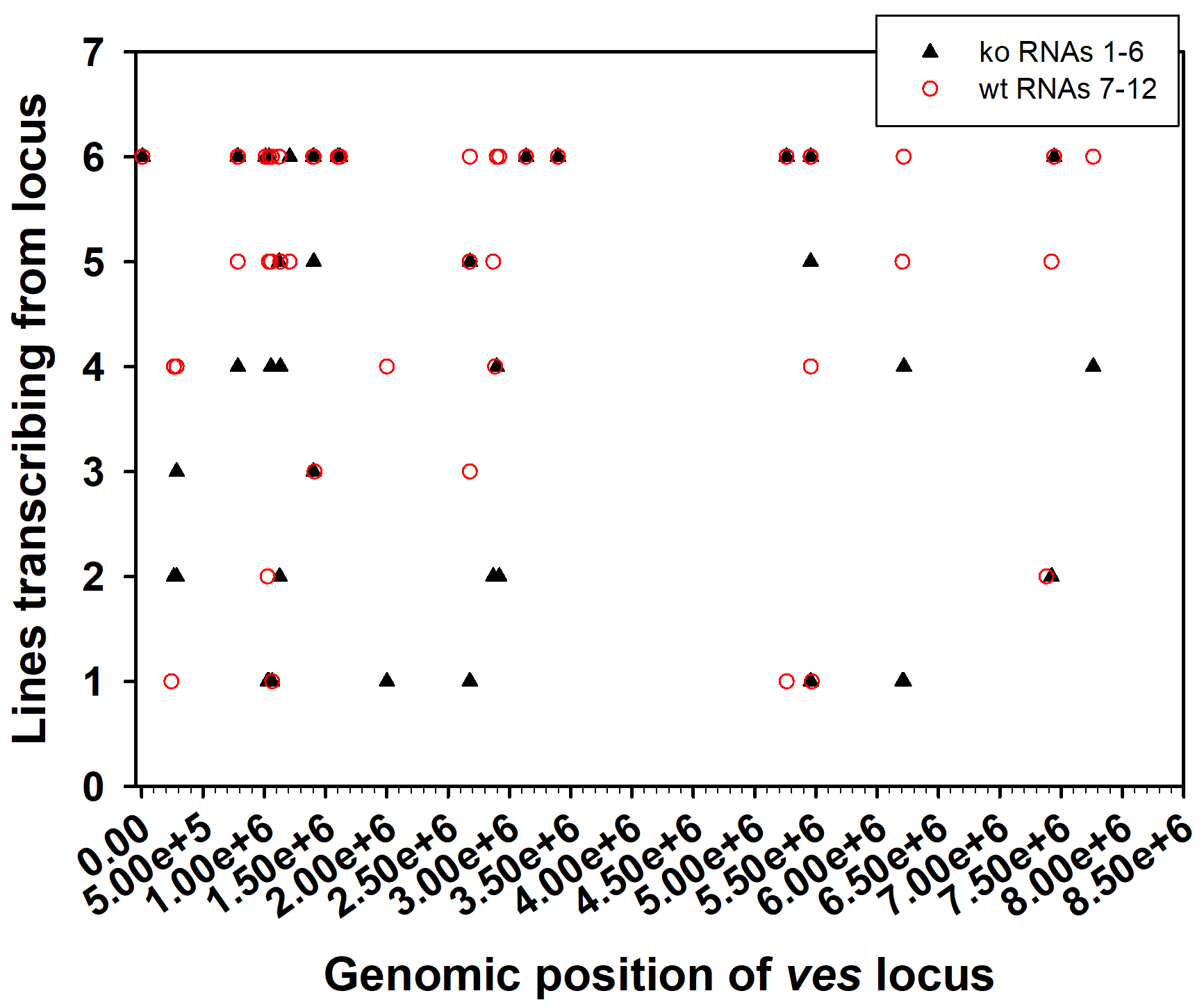

Supplement: S2 Fig — The genomic locations of ves loci to which amplicons mapped (plotting the full 8 Mbp genome as a single linear element), and from which they were presumed to be transcribed, is plotted relative to the numbers of parasite lines transcribing from that locus. No ves gene clusters within the genome were transcribed by CE11 parasites that could not also be transcribed by knockout parasites, and vice versa. For this plot, data were pooled from both time-points of all three wt or all three knockout lines (i.e., maximally 6 possible per ves locus). (TIF) [file ppat.1008772.s002.tif]

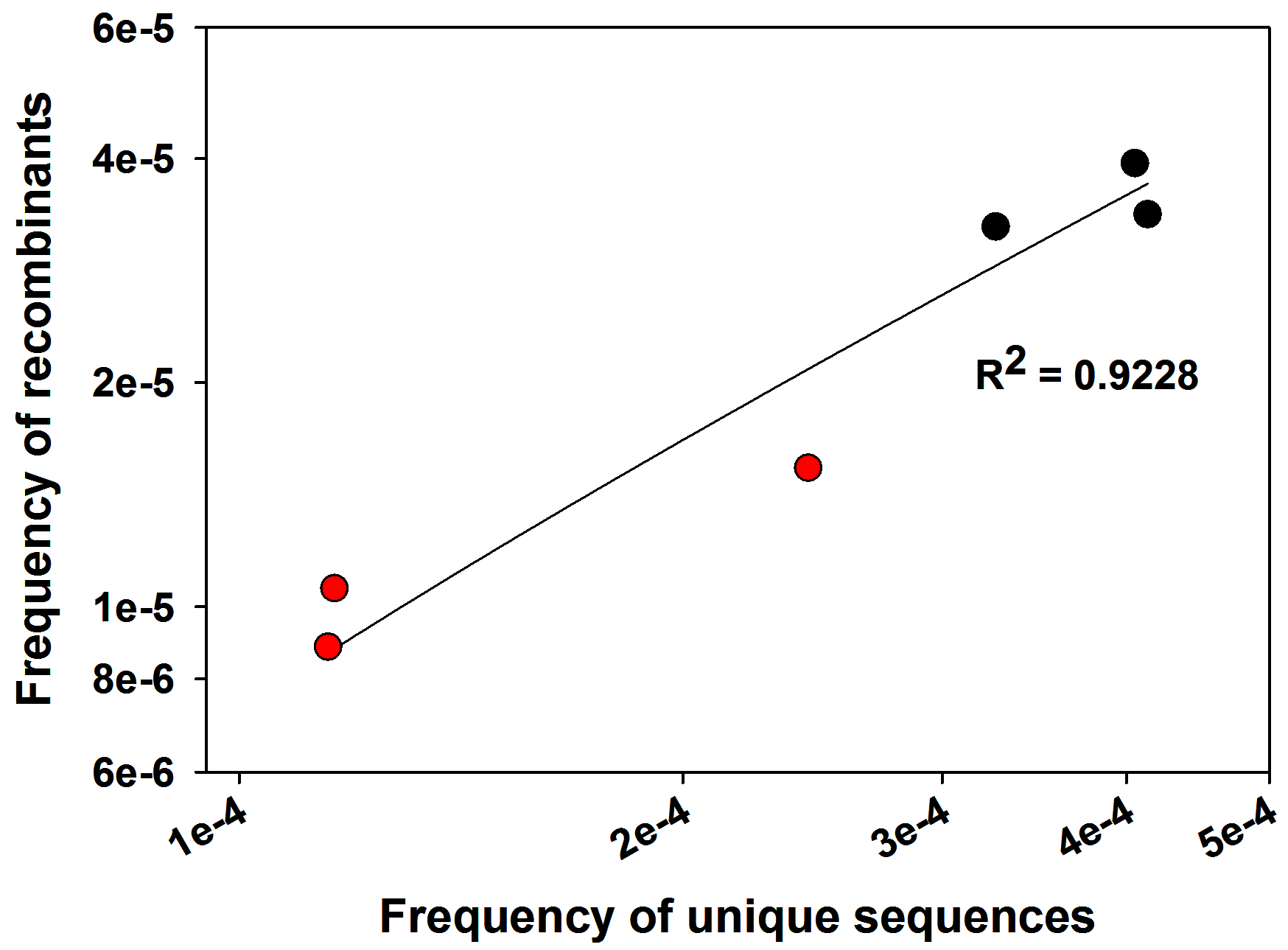

Supplement: S3 Fig — A regression plot was made of the frequencies of merged amplicons with full statistical support for identification as true recombinants against the total numbers of unique amplicons observed for each population, with each type of value normalized per million reads (R2 = 0.9228). (TIF) [file ppat.1008772.s003.tif]

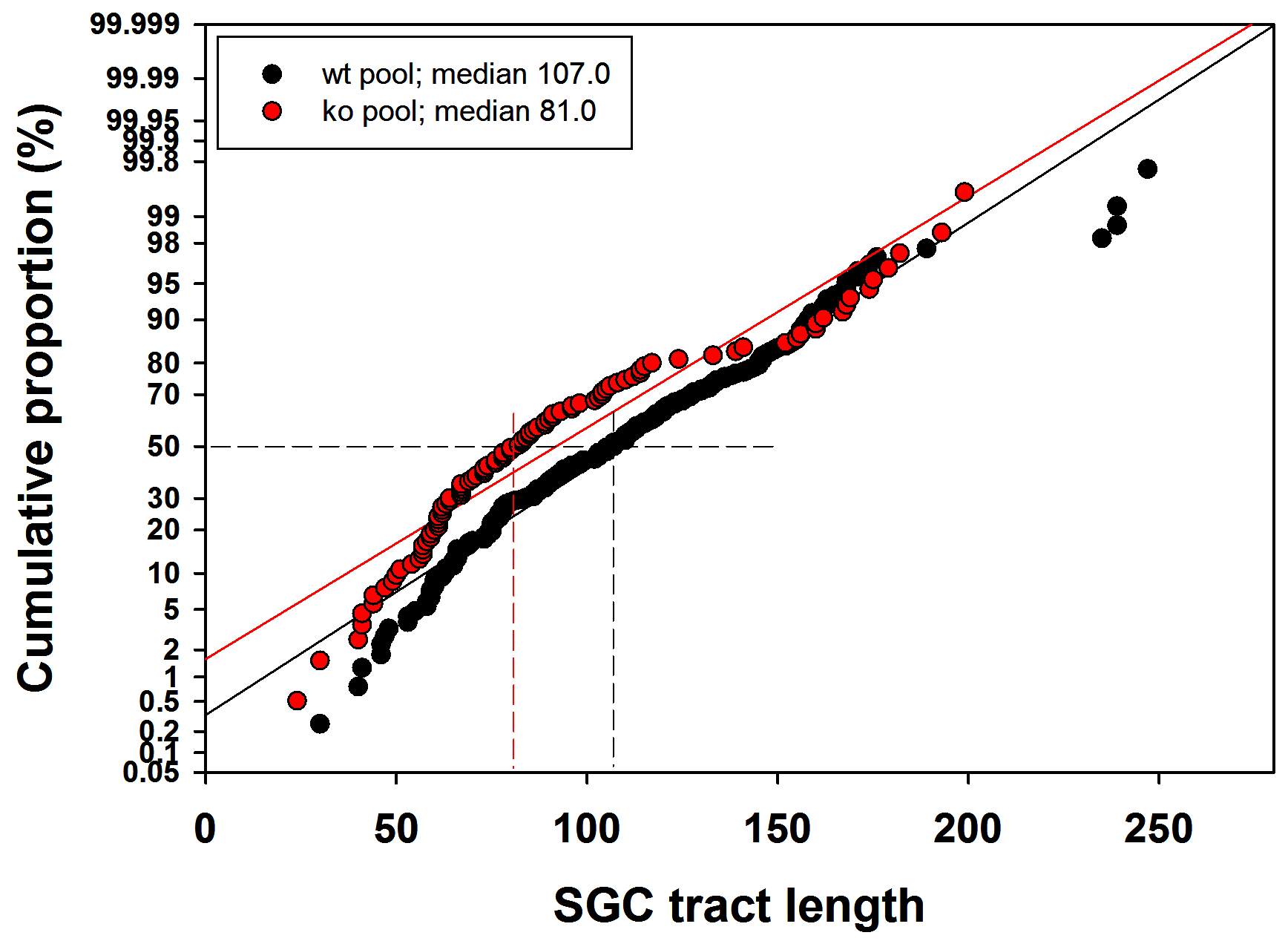

Supplement: S4 Fig — The length distribution of unique SGC tracts was plotted as the cumulative proportion of total SGC tracts against tract lengths. Samples were pooled for all six samples for wild-type and knockout parasites. These pooled data are from the same CE11 wild-type and knockout lines shown in Fig 4B. The vertical index lines indicates the median values of each population. Differences among the distributions in each sample type were determined by the Mann-Whitney Rank Sum test (p = <0.001). (TIF) [file ppat.1008772.s004.tif]

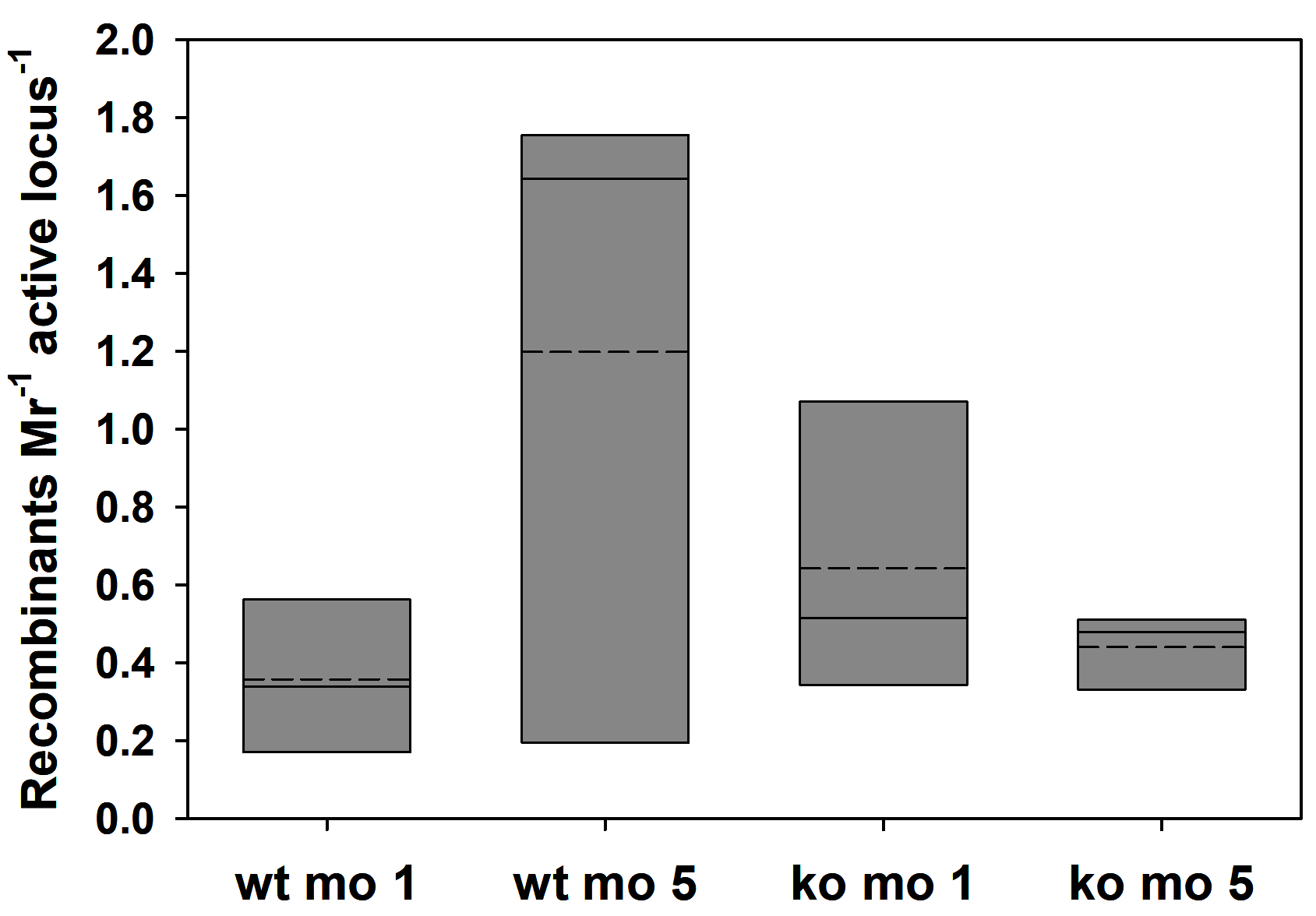

Supplement: S5 Fig — The numbers of unique SGC tracts with full statistical support per transcriptionally active ves locus is shown for B. bovis CE11 wild-type and knockout populations at 1 and 5 months growth post-cloning. The data were pooled from all three lines of each population type. Slotted crossbars indicate means, solid crossbars indicate medians, and the box boundaries represent 25% and 75% confidence intervals. Values did not vary significantly among samples (p = 0.986, based upon one-way ANOVA). (TIF) [file ppat.1008772.s005.tif]
