# Supplementary material for: Babesia bovis Rad51 ortholog influences switching of ves genes but is not essential for segmental gene conversion in antigenic variation
Source: PLoS Pathog. 2020 Aug 31;16(8):e1008772. doi: 10.1371/journal.ppat.1008772 (PMC7485966; doi:10.1371/journal.ppat.1008772)
Supplement: S1 Table — Loci to which amplicons mapped are listed for all 12 samples (i.e., 1-month and 5-month RNAs from all three wild-type and knockout lines), along with mean lengths and the nature of each locus. Both divergent, bidirectional loci and unidirectional loci were active in transcription at some level in all samples. (DOCX) [file ppat.1008772.s006.docx]

**S1 Table. Organizational nature of the *ves* loci to which reads mapped.** These mappings are based upon the *Babesia bovis* C9.1 line genome (available at ftp://ftp.sanger.ac.uk/pub/pathogens/Babesia/).

**RNA1: ko1, 1 month**

Region p-value^1^ Length^2^ Locus type^3^

6915-7268 0.00 354 divergent a/a^4^

782938-783288 0.00 351 unidirectional a

786356-786709 0.00 354 unidirectional a

1011139-1011492 0.00 354 unidirectional a

1039551-1039898 0.00 348 unidirectional a

1055223-1055551 0.00 329 divergent b^4^/a

1122014-1122364 0.00 351 divergent a/a

1208548-1208877 0.00 330 divergent a/b

1399269-1399601 0.00 333 divergent b/a

1403602-1403967 0.00 366 divergent b/a

1404548-1404874 0.00 327 divergent b/a

1600915-1601241 0.00 327 unidirectional a

1618150-1618599 0.00 450 unidirectional a

2678070-2678405 0.00 336 divergent a/a

3137303-3137650 0.00 348 no annotated gene

3396845-3397184 0.00 340 no annotated gene

5260614-5260935 0.00 322 divergent b/a

5456956-5457291 0.00 336 divergent b/a

5458987-5459181 0.00 195 divergent b/a

6205332-6205667 0.00 336 unidirectional a

6218668-6219009 0.00 342 divergent a/a

7443575-7443907 0.00 333 unidirectional a

7762155-7762487 0.00 333 divergent a/b

10 divergent; 8 unidirectional; 2 with no annotated gene (20 total); div/uni= 1.25.

**RNA2: ko1, 5 months**

Region p-value Length Locus type

6915-7268 0.00 354 divergent a/a

285751-286083 0.00 333 divergent a/a

782938-783288 0.00 351 unidirectional a

785282-785626 0.00 345 unidirectional a

786356-786709 0.00 354 unidirectional a

1011139-1011492 0.00 354 unidirectional a

1037935-1038261 0.00 327 unidirectional a

1039551-1039898 0.00 348 unidirectional a

1055223-1055551 0.00 329 divergent b/a

1122014-1122364 0.00 351 divergent a/a

1133046-1133396 0.00 351 divergent b/a

1208548-1208877 0.00 330 divergent a/b

1399269-1399601 0.00 333 divergent b/a

1403602-1403967 0.00 366 divergent b/a

1404548-1404874 0.00 327 divergent b/a

1600915-1601241 0.00 327 unidirectional a

1618150-1618599 0.00 450 unidirectional a

2678070-2678405 0.00 336 divergent ves/ves

2867318-2867671 0.00 354 divergent a/b

2897991-2898213 8.55E-7 223 unidirectional a

3137303-3137650 0.00 348 no annotated gene

3396845-3397184 0.00 340 no annotated gene

5260614-5260991 0.00 378 divergent b/a

5456956-5457291 0.00 336 divergent b/a

5458987-5459181 0.00 195 divergent b/a

6218668-6219009 0.00 342 divergent a/a

7443575-7443907 0.00 333 unidirectional a

7762155-7762487 0.00 333 divergent a/b

13 divergent; 7 unidirectional; 2 no annotated gene (22 total); div/uni= 1.86

**RNA3: ko2, 1 month**

Region p-value Length Locus type

6915-7268 0.00 354 divergent a/a

782938-783288 0.00 351 unidirectional a

786356-786709 0.00 354 unidirectional a

1011139-1011492 0.00 354 unidirectional a

1039551-1039898 0.00 348 unidirectional a

1064683-1065007 0.00 325 divergent a/b

1133046-1133396 0.00 351 divergent b/a

1208548-1208877 0.00 330 divergent a/b

1399269-1399601 0.00 333 divergent b/a

1402278-1402513 5.49E-15 236 divergent b/a

1403602-1403967 0.00 366 divergent b/a

1600915-1601241 0.00 327 unidirectional a

1618150-1618599 0.00 450 unidirectional a

2678070-2678405 0.00 336 divergent ves/ves

2678790-2678964 9.73E-7 175 divergent ves/ves

3137303-3137650 0.00 348 no annotated gene

3396845-3397184 0.00 340 no annotated gene

5260614-5260991 6.61E-7 378 divergent b/a

5457143-5457291 0.00 149 divergent b/a

5458987-5459181 0.00 195 divergent b/a

6218685-6218996 1.92E-7 312 divergent a/a

7443575-7443907 0.00 333 unidirectional a

7762155-7762487 0.00 333 divergent a/b

10 divergent; 6 unidirectional; 2 no annotated gene (20 total); div/uni= 1.67

**RNA4: ko2, 5 months**

Region p-value Length Locus type

6915-7268 3.52E-15 354 divergent a/a

264245-264601 0.00 357 unidirectional a

286923-287037 0.00 115 divergent a/a

782938-783288 0.00 351 unidirectional a

785381-785471 2.17E-8 91 unidirectional a

786356-786709 0.00 354 unidirectional a

1011139-1011492 0.00 354 unidirectional a

1039551-1039898 0.00 348 unidirectional a

1055223-1055551 0.00 329 divergent b/a

1122014-1122364 0.00 351 divergent a/a

1125758-1125858 1.76E-15 101 divergent ves/a

1208548-1208877 0.00 330 divergent a/b

1399269-1399601 0.00 333 divergent b/a

1402277-1402513 4.81E-9 237 divergent b/a

1403602-1403967 0.00 366 divergent b/a

1404663-1404777 7.26E-7 115 divergent b/a

1600915-1601241 0.00 327 unidirectional a

1618150-1618599 0.00 450 unidirectional a

2897979-2898313 0.00 335 unidirectional a

2918405-2918586 0.00 182 divergent b/ves

3137303-3137650 0.00 348 no annotated gene

3396845-3397184 0.00 340 no annotated gene

5260614-5260991 0.00 378 divergent b/a

5457143-5457291 5.16E-14 149 divergent b/a

5458986-5459181 0.00 196 divergent b/a

7421824-7421883 6.72E-8 60 unidirectional a

7443575-7443907 0.00 333 unidirectional a

7762155-7762487 0.00 333 divergent a/b

12 divergent; 9 unidirectional; 2 no annotated gene (23 total); div/uni= 1.33

**RNA5: ko3, 1 month**

Region p-value Length Locus type

6915-7268 0.00 354 divergent a/a

264245-264601 0.00 357 unidirectional a

285770-286071 7.21E-7 302 divergent a/a

286759-287112 1.90E-7 354 divergent a/a

782938-783288 0.00 351 unidirectional a

785282-785626 0.00 345 unidirectional a

786375-786585 9.81E-6 211 unidirectional a

1011139-1011492 0.00 354 unidirectional a

1028822-1029096 0.00 275 divergent b/a

1039551-1039898 0.00 348 unidirectional a

1122014-1122364 0.00 351 divergent a/a

1133046-1133396 0.00 351 divergent b/a

1208548-1208877 0.00 330 divergent a/b

1399269-1399601 0.00 333 divergent b/a

1403602-1403967 0.00 366 divergent b/a

1404548-1404874 0.00 327 divergent b/a

1600915-1601241 7.98E-14 327 unidirectional a

1618168-1618580 2.83E-6 413 unidirectional a

2678070-2678394 6.75E-10 325 divergent ves/ves

2867329-2867671 8.14E-7 343 divergent a/b

2897979-2898332 0.00 354 unidirectional a

3137303-3137650 0.00 348 no annotated gene

3396845-3397186 0.00 342 no annotated gene

5260614-5260935 3.86E-17 322 divergent b/a

5457143-5457291 0.00 149 divergent b/a

5458074-5458335 0.00 262 divergent b/a

5458987-5459181 0.00 195 divergent b/a

6216891-6217207 6.83E-8 317 divergent a/a

6218668-6219009 0.00 342 divergent a/a

7443575-7443907 0.00 333 unidirectional a

7762166-7762487 1.24E-9 322 divergent a/b

16 divergent; 8 unidirectional; 2 no annotated gene (26 total); div/uni= 2.00

**RNA6: ko3, 5 months**

Region p-value Length Locus type

6915-7268 0.00 354 divergent a/a

286759-287112 0.00 354 divergent a/a (a pair)

782938-783288 0.00 351 unidirectional a

785379-785471 2.08E-11 93 unidirectional a

786356-786709 0.00 354 unidirectional a

1011139-1011492 0.00 354 unidirectional a

1039551-1039898 0.00 348 unidirectional a

1055223-1055551 0.00 329 divergent b/a

1122014-1122364 0.00 351 divergent a/a

1125758-1125858 0.00 101 divergent a/a

1133046-1133396 0.00 351 divergent b/a

1208548-1208877 0.00 330 divergent a/b

1399269-1399601 0.00 333 divergent b/a

1402278-1402513 0.00 236 divergent b/a

1403602-1403967 0.00 366 divergent b/a

1404548-1404874 0.00 327 divergent b/a

1600915-1601241 0.00 327 unidirectional a

1618150-1618599 0.00 450 unidirectional a

2001903-2002139 0.00 237 unidirectional a

2676785-2676969 0.00 185 divergent ves/ves

2678070-2678405 1.45E-10 336 divergent a/a

2897979-2898313 5.69E-14 335 unidirectional a

2918405-2918586 0.00 182 divergent b/ves

3137303-3137650 1.60E-9 348 no annotated gene

3396845-3397184 0.00 340 no annotated gene

5260614-5260991 0.00 378 divergent b/a

5458986-5459181 2.06E-16 196 divergent b/a

7421824-7421883 1.57E-12 60 unidirectional a

7443575-7443907 0.00 333 unidirectional a

7762155-7762487 0.00 333 divergent a/b

14 divergent; 9 unidirectional; 2 no annotated gene (25 total); div/uni= 1.56

**RNA7: CE11(B8), 1 month**

Region p-value Length Locus type

6915-7268 0.00 354 divergent a/a

264245-264601 6.37E-9 357 unidirectional a

782938-783288 0.00 351 unidirectional a

785282-785626 0.00 345 unidirectional a

786356-786709 0.00 354 unidirectional a

1011139-1011492 0.00 354 unidirectional a

1028822-1029096 0.00 275 divergent b/a

1037935-1038261 3.73E-18 327 unidirectional a

1039551-1039898 0.00 348 unidirectional a

1055223-1055551 0.00 329 divergent b/a

1064683-1065007 1.33E-15 325 divergent a/b

1122014-1122364 0.00 351 divergent a/a

1133046-1133396 0.00 351 divergent b/a

1208548-1208877 0.00 330 divergent a/b

1399269-1399601 0.00 333 divergent b/a

1402184-1402513 0.00 330 divergent b/a

1403602-1403967 0.00 366 divergent b/a

1404548-1404874 0.00 327 divergent b/a

1411481-1411704 0.00 224 unidirectional a

1600915-1601241 0.00 327 unidirectional a

1618150-1618599 0.00 450 unidirectional a

2001876-2002139 0.00 264 unidirectional a

2676785-2676969 0.00 185 divergent ves/ves

2678070-2678405 0.00 336 divergent a/a

2867318-2867671 0.00 354 divergent a/b

2882658-2882975 4.26E-10 318 unidirectional a

2897979-2898332 0.00 354 unidirectional a

2918405-2918586 0.00 182 divergent b/ves

3137303-3137650 0.00 348 no annotated gene

3396845-3397186 1.17E-16 342 no annotated gene

5260614-5260991 0.00 378 divergent b/a

5456956-5457291 0.00 336 divergent b/a

5458074-5458335 0.00 262 divergent b/a

5458922-5459254 0.00 333 divergent b/a

6205332-6205667 0.00 336 unidirectional a

6218679-6218996 8.82E-10 318 divergent a/a

7380075-7380237 2.37E-7 163 unidirectional a

7421547-7421883 0.00 337 unidirectional a

7443575-7443907 0.00 333 unidirectional a

7762155-7762487 0.00 333 divergent a/b

16 divergent; 15 unidirectional; 2 no annotated gene (33 total); div/uni= 1.07

**RNA8: CE11(B8), 5 months**

Region p-value Length Locus type

6915-7268 0.00 354 divergent a/a

264245-264601 5.02E-7 357 unidirectional a

782938-783288 0.00 351 unidirectional a

785282-785626 0.00 345 unidirectional a

786356-786709 0.00 354 unidirectional a

1011139-1011492 0.00 354 unidirectional a

1028822-1029096 0.00 275 divergent b/a

1037946-1038261 1.39E-7 316 unidirectional a

1039551-1039898 0.00 348 unidirectional a

1055223-1055551 0.00 329 divergent b/a

1064683-1065007 0.00 325 divergent a/b

1122014-1122364 0.00 351 divergent a/a

1133046-1133396 0.00 351 divergent b/a

1208548-1208877 0.00 330 divergent a/b

1399269-1399601 0.00 333 divergent b/a

1402184-1402513 0.00 330 divergent b/a

1403602-1403967 0.00 366 divergent b/a

1404548-1404874 0.00 327 divergent b/a

1411481-1411704 0.00 224 unidirectional a

1600915-1601241 0.00 327 unidirectional a

1618150-1618599 0.00 450 unidirectional a

2001876-2002139 0.00 264 unidirectional a

2676785-2676969 0.00 185 divergent ves/ves

2678070-2678405 0.00 336 divergent a/a

2867318-2867671 0.00 354 divergent a/b

2882658-2882975 7.82E-13 318 unidirectional a

2897979-2898332 0.00 354 unidirectional a

2918405-2918586 0.00 182 divergent b/ves

3137303-3137650 0.00 348 no annotated gene

3396845-3397184 0.00 340 no annotated gene

5260614-5260991 0.00 378 divergent b/a

5456956-5457291 0.00 336 divergent b/a

5458074-5458335 0.00 262 divergent b/a

5458922-5459254 0.00 333 divergent b/a

6205332-6205667 0.00 336 unidirectional a

6218672-6218996 9.92E-7 325 divergent a/a

7380075-7380237 1.15E-8 163 unidirectional a

7421547-7421883 0.00 337 unidirectional a

7443575-7443907 0.00 333 unidirectional a

7762155-7762487 0.00 333 divergent a/b

16 divergent; 15 unidirectional; 2 no annotated gene (33 total); div/uni= 1.07

**RNA9: CE11(C2), 1 month**

Region p-value Length Locus type

6915-7268 0.00 354 divergent a/a

264245-264601 0.00 357 unidirectional a

782938-783288 0.00 351 unidirectional a

786356-786709 0.00 354 unidirectional a

1011139-1011492 0.00 354 unidirectional a

1037954-1038243 1.21E-6 290 unidirectional a

1039551-1039898 0.00 348 unidirectional a

1064683-1065007 8.19E-11 325 divergent a/b

1122014-1122364 0.00 351 divergent a/a

1208548-1208877 0.00 330 divergent a/b

1399269-1399601 0.00 333 divergent b/a

1402278-1402513 5.17E-18 236 divergent b/a

1403602-1403967 0.00 366 divergent b/a

1404548-1404874 0.00 327 divergent b/a

1600915-1601241 0.00 327 unidirectional a

1618161-1618580 1.15E-7 420 unidirectional a

2678070-2678405 0.00 336 divergent a/a

2882677-2882964 4.20E-10 288 unidirectional a

2897979-2898321 3.61E-8 343 unidirectional a

2918405-2918586 4.28E-9 182 divergent b/ves

3137303-3137650 0.00 348 no annotated gene

3396845-3397184 0.00 340 no annotated gene

5260614-5260991 0.00 378 divergent b/a

5456956-5457291 0.00 336 divergent b/a

5458074-5458335 0.00 262 divergent b/a

5458987-5459181 0.00 195 divergent b/a

6205332-6205667 0.00 336 unidirectional a

6216872-6217207 1.72E-8 336 divergent a/a

6218668-6219009 0.00 342 divergent a/a (pair)

7443575-7443907 0.00 333 unidirectional a

7762155-7762487 0.00 333 divergent a/b

12 divergent; 11 unidirectional; 2 no annotated gene (25 total); div/uni= 1.09

**RNA10: CE11(C2), 5 months**

Region p-value Length Locus type

6915-7268 0.00 354 divergent a/a

243324-243551 0.00 228 unidirectional a

264245-264601 0.00 357 unidirectional a

285751-286083 0.00 333 divergent a/a

286759-287112 0.00 354 divergent a/a (a pair)

782938-783288 0.00 351 unidirectional a

785282-785626 0.00 345 unidirectional a

786356-786709 0.00 354 unidirectional a

1011139-1011492 0.00 354 unidirectional a

1037935-1038261 0.00 327 unidirectional a

1039551-1039898 0.00 348 unidirectional a

1055223-1055551 0.00 329 divergent b/a

1064702-1064889 2.81E-6 188 divergent a/b

1065065-1065243 0.00 179 divergent a/b

1122014-1122364 0.00 351 divergent a/a

1125759-1125858 5.81E-7 100 divergent a/a (a pair)

1133046-1133396 0.00 351 divergent b/a

1208548-1208877 0.00 330 divergent a/b

1399269-1399601 0.00 333 divergent b/a

1402184-1402513 6.20E-14 330 divergent b/a

1403602-1403967 0.00 366 divergent b/a

1404548-1404874 0.00 327 divergent b/a

1600915-1601241 0.00 327 unidirectional a

1618150-1618599 0.00 450 unidirectional a

2001903-2002139 5.05E-7 237 unidirectional a

2676785-2676969 0.00 185 divergent ves/ves

2678070-2678405 0.00 336 divergent a/a

2678837-2678963 2.69E-6 127 divergent ves/ves

2867318-2867671 0.00 354 divergent a/b

2897979-2898332 0.00 354 unidirectional a

2918405-2918586 0.00 182 divergent b/ves

3137303-3137650 0.00 348 no annotated gene

3396845-3397184 0.00 340 no annotated gene

5260614-5260991 0.00 378 divergent b/a

5262801-5263089 0.00 289 divergent b/a

5456956-5457291 0.00 336 divergent b/a

5458074-5458368 0.00 295 divergent b/a

5458922-5459254 0.00 333 divergent b/a

5467192-5467427 4.61E-14 236 divergent b/a

6205332-6205667 0.00 336 unidirectional a

6218668-6219009 0.00 342 divergent a/a

7421547-7421883 0.00 337 unidirectional a

7443575-7443907 0.00 333 unidirectional a

7762155-7762487 0.00 333 divergent a/b

20 divergent; 13 unidirectional; 2 with no annotated gene (35 total); div/uni= 1.54

**RNA11: CE11(C5), 1 month**

Region p-value Length Locus type

6915-7268 0.00 354 divergent a/a

286759-287112 0.00 354 divergent a/a

782938-783288 0.00 351 unidirectional a

785282-785626 0.00 345 unidirectional a

786356-786709 0.00 354 unidirectional a

1011139-1011492 0.00 354 unidirectional a

1039551-1039898 0.00 348 unidirectional a

1055223-1055551 0.00 329 divergent b/a

1064683-1065243 0.00 561 divergent a/b

1122014-1122364 0.00 351 divergent a/a

1133046-1133396 0.00 351 divergent b/a

1399269-1399601 0.00 333 divergent b/a

1402277-1402513 2.05E-7 237 divergent b/a

1403602-1403967 0.00 366 divergent b/a

1404548-1404874 0.00 327 divergent b/a

1600915-1601241 0.00 327 unidirectional a

1618150-1618599 0.00 450 unidirectional a

2676785-2676969 0.00 185 divergent ves/ves

2678070-2678405 0.00 336 divergent a/a

2678755-2678964 2.35E-7 210 divergent ves/ves

2867318-2867671 0.00 354 divergent a/b

2882658-2882975 0.00 318 unidirectional a

2897990-2898213 1.76E-8 224 unidirectional a

2918405-2918586 2.29E-7 182 divergent b/ves

3137303-3137650 0.00 348 no annotated gene

3396845-3397184 0.00 340 no annotated gene

5260614-5260991 0.00 378 divergent b/a

5456956-5457291 0.00 336 divergent b/a

5458987-5459181 0.00 195 divergent b/a

6218668-6219009 0.00 342 divergent a/a

7421547-7421883 0.00 337 unidirectional a

7443575-7443907 0.00 333 unidirectional a

7762155-7762487 0.00 333 divergent a/b

16 divergent; 10 unidirectional; 2 no annotated gene (28 total); div/uni= 1.60

**RNA12: CE11(C5), 5 months**

Region p-value Length Locus type

6915-7268 0.00 354 divergent a/a

285751-286083 0.00 333 divergent a/a

782938-783288 0.00 351 unidirectional a

785282-785626 0.00 345 unidirectional a

786356-786709 0.00 354 unidirectional a

1011139-1011492 0.00 354 unidirectional a

1037935-1038261 0.00 327 unidirectional a

1039384-1039539 7.17E-6 156 unidirectional a

1039551-1039898 0.00 348 unidirectional a

1055223-1055551 0.00 329 divergent b/a

1064683-1065243 0.00 561 divergent a/b

1122014-1122364 0.00 351 divergent a/a

1133046-1133396 0.00 351 divergent b/a

1208548-1208877 0.00 330 divergent a/b

1399269-1399601 0.00 333 divergent b/a

1402278-1402513 0.00 236 divergent b/a

1403602-1403967 0.00 366 divergent b/a

1404548-1404874 0.00 327 divergent b/a

1411516-1411704 0.00 189 unidirectional a

1600915-1601241 0.00 327 unidirectional a

1618150-1618599 0.00 450 unidirectional a

2001961-2002139 0.00 179 unidirectional a

2676785-2676969 0.00 185 divergent ves/ves

2678070-2678405 0.00 336 divergent a/a

2678770-2678964 2.51E-7 195 divergent ves/ves

2867318-2867671 0.00 354 divergent a/b

2897979-2898332 0.00 354 unidirectional a

2918405-2918586 0.00 182 divergent b/ves

3137303-3137650 0.00 348 no annotated gene

3396845-3397184 0.00 340 no annotated gene

5260614-5260991 0.00 378 divergent b/a

5456956-5457291 0.00 336 divergent b/a

5458987-5459181 0.00 195 divergent b/a

6205332-6205667 0.00 336 unidirectional a

6218668-6219009 0.00 342 divergent a/a

7421547-7421883 0.00 337 unidirectional a

7443575-7443907 0.00 333 unidirectional a

7761921-7762129 0.00 209 divergent a/b

7762155-7762487 0.00 333 divergent a/b

17 divergent; 13 unidirectional; 2 no annotated gene (32 total); div/uni= 1.31

^1^”p-value” indicates the statistical value for the alignment of the mapped sequence with that of the genomic locus to which it mapped.

^2^”Length” refers to the merged length of the mapped amplicons, in base pairs. This is shorter than the lengths of the primary amplicons because of having trimmed primer sequences from the ends.

^3^”Locus type” indicates whether the *ves* locus to which an amplicon mapped was comprised of a single *ves* gene (unidirectional), a *ves* gene in a divergent pair (divergent), or was one that was not annotated.

^4^”a”, “b”, “a/a”, “a/b”, and “b/a” refer to *ves* α, *ves* β, and pairs of *ves* genes in α/α or α/β pairs, and the order of the genes relative to genomic position. Underlining indicates the member of a gene pair to which an amplicon mapped.

Red, boxed sequences indicate *ves* loci which were assembled with multiple CKRD domains, and to which multiple amplicons mapped.
