# Supplementary material for: Babesia bovis Rad51 ortholog influences switching of ves genes but is not essential for segmental gene conversion in antigenic variation
Source: PLoS Pathog. 2020 Aug 31;16(8):e1008772. doi: 10.1371/journal.ppat.1008772 (PMC7485966; doi:10.1371/journal.ppat.1008772)
Supplement: Attachment — Submitted filename: MackEA_response_to_reviewers.docx [file ppat.1008772.s008.docx]

**General comments**:

*Reviewer 1*

*“Is there a potential for a mixture of DNA recombination pathways contributing to the ves gene diversification and should be referred to differently?”* We agree, and now mention that some of the changes we have observed may reflect the operation of more than one mechanism. However, we do not specifically propose this, as we feel that would be extrapolating well beyond what is actually supported by the data. We also agree that segmental gene conversion may be a misnomer, given the nature of recombination we observed. However, without actual evidence of what other mechanism(s) is/are occurring, we continue to refer to this as SGC for historical reasons until more is known.

*“..there may be some potential bias in their approach in that they may have missed larger recombination events”*. We agree, and now mention this at lines 436-439, but also attempt to outline some of the difficulties in actually looking at both the fine and larger-scale events simultaneously (lines 422-425). However, this does not diminish the value of looking closely at the SGC event itself in a targeted manner. Moreover, at the time much of this work was done, some currently available methodologies did not yet exist.

*Reviewer 2*: none requiring a response.

**Major Issues**:

*Reviewer 1*: none.

*Reviewer 2*

*“I’m afraid that I am not certain what we have learned from the first set of experiments described…”*. Based upon the reviewer’s concerns that the genetic fusion of the Bb*rad51* gene with the *glmS* element may artifactually alter BbRad51 levels and/or activity, and the limited additional understanding that would be provided by that experiment even without caveats, we have eliminated it from the manuscript. This option was suggested by the Editor. Accordingly, we also have deleted lines 160-190, 572-582, Figure 1, and S2 and S3 Files of the original manuscript that were associated with this experiment.

**Minor Issues**

*Reviewer 1*

*“I would think that Figues 5, 6 and 7 could be combined though it would create a long figure legend. Either way, if possible Supplemental Fig 7 should be part of the main body of the paper.”* In response to the reviewer’s suggestion, we have combined Figures 5 and 6 into a new Figure 4A and B, demonstrating effectively the processivity and frequency of initiation of the SGC process. However, Figure 7 and S7 Figure effectively deal with the nature of the sequences involved in the junctional aspects of the recombination process (or perhaps its aftermath). We felt they could more logically be combined into a single Figure 5A and B, bringing S7 Figure within the main body of the manuscript as suggested, but reducing the overall number of figures.

*“Page 10 line 279- can you clarify why not feasible? Perhaps rephrase”*. We have rewritten this (new lines 250-257) as, “…, but this approach has its own limitations. Given that such a genome relies upon consistent sequence this would require cloning of the parasite, effectively selecting one variant from a pool of possible variants. This would bias the outcome toward apparently greater or lesser similarity depending upon how representative the cloned parasite is of the major population. One potential approach to solve this difficulty might be to use conserved *ves* family sequence patches [12] to perform a targeted sequence enrichment prior to library construction, followed by deep sequencing [34].”

*“Page 12 line 336 – typo”*. The typo has been corrected.

*“page 15 line 436- did you mean supp fig7?”*. We have clarified that this refers to the new Figure 5.

*“Page 15 line 437- the authors humbly acknowledge the limitations of their study. It would be nice to see a follow up where they state how they would/will address in the future perhaps with WGS.”* We now address this more directly, including problems with WGS, in new lines 250-257 and 422-425.

*Reviewer 2*

2. *“To my mind the title of the section ‘Bbrad51 knockout did not prevent in situ transcriptional switching’ is misleading.”* We have retitled this as, “Bb*rad51* knockout resulted in apparent reduction of in situ transcriptional switching”, in order to suggest broader effects.

3. *“I would suggest that a diagram summarising how the authors have assayed SGC is needed, as this is very hard to determine from results and methods, and is important for the reader to understand the data.”*. We now provide a schematic diagram (new Figure 3) outlining the flow of the experiment, in order to make it easier for a reader to understand how the experiment was done.

4. *“Again, I think that the title of the section ‘Bbrad51 knockout failed to prevent segmental gene conversion’ is misleading (or describes only part of the data).”*. We have retitled this section as, “Bb*rad51* knockout reduces overall variation without abrogating segmental gene conversion”, again in order to suggest broader outcomes.

5. *“The concern in point 4 filters into the discussion: e.g. line 429 ‘When initiating this study we hypothesized that, as Rad51 proteins are considered essential to HR and SGC is thought to be a form of HR, BbRad51 should be essential to this process and its loss should abrogate SGC. Our results disprove that hypothesis’.”* Whereas we feel our original statement to be factually correct, in order to be more inclusive of overall outcome and to avoid a heavy-handed conclusion we have rewritten this as, “Our results disprove our hypothesis of essentiality, but do not preclude a contribution by BbRad51 to the process.” We also have heavily rewritten and slightly expanded the discussion of this section, in new lines 407-436.

6. *“In line 382 where in ‘this study’ is it documented that Ku and Ligase 4 are absent in Babesia?”*. We apologize for this error. “..this study” was a stray leftover from a previous form of this manuscript, and has been deleted.

7. *“The change in in situ transcription in the rad51 mutants described in this work is intriguing and well*

*discussed. Has a similar effect been described in any other pathogen that relies on transcription for antigenic variation, such as Plasmodium or Trypanosoma? In other words, is this finding likely to have implications beyond Babesia?”*. Thank you for this opportunity to speculate on the broader significance of our findings, as we have also pondered the question expressed by the reviewer. We have now added (new lines 508-514), “It is noteworthy that these results are similar to observations in *T. brucei*, which possesses five Rad51 protein family genes. Knockouts of Tb*Rad51* and Tb*Rad51-3* each were shown to result in diminution of *VSG* gene switch events not dependent upon DNA recombination, as well as the expected HR-associated reductions [28, 54]. This observation suggests that such proposed interactions of Rad51 proteins with the epigenetic machinery may be widespread phylogenetically, but not yet widely appreciated.”
